# Sleep-Dependent Synaptic Down-Selection (I): Modeling the Benefits of Sleep on Memory Consolidation and Integration

**DOI:** 10.3389/fneur.2013.00143

**Published:** 2013-09-30

**Authors:** Andrew Nere, Atif Hashmi, Chiara Cirelli, Giulio Tononi

**Affiliations:** ^1^Department of Electrical and Computer Engineering, University of Wisconsin-Madison, Madison, WI, USA; ^2^Department of Psychiatry, University of Wisconsin-Madison, Madison, WI, USA

**Keywords:** neurons, plasticity and learning, sleep, homeostatic regulation, declarative memory, procedural memory

## Abstract

Sleep can favor the consolidation of both procedural and declarative memories, promote gist extraction, help the integration of new with old memories, and desaturate the ability to learn. It is often assumed that such beneficial effects are due to the reactivation of neural circuits in sleep to further strengthen the synapses modified during wake or transfer memories to different parts of the brain. A different possibility is that sleep may benefit memory not by further strengthening synapses, but rather by renormalizing synaptic strength to restore cellular homeostasis after net synaptic potentiation in wake. In this way, the sleep-dependent reactivation of neural circuits could result in the competitive down-selection of synapses that are activated infrequently and fit less well with the overall organization of memories. By using computer simulations, we show here that synaptic down-selection is in principle sufficient to explain the beneficial effects of sleep on the consolidation of procedural and declarative memories, on gist extraction, and on the integration of new with old memories, thereby addressing the plasticity-stability dilemma.

## 1 Introduction

Sleep has positive effects on memory consolidation, as shown by various experimental paradigms in which newly formed memories are preserved better after a night of sleep than after an equivalent amount of time spent awake. This benefit is well documented for declarative memories – those one can recollect consciously, such as lists of words or associations between pictures and places (1–7). Non-declarative memories, such as perceptual and motor skills, can also profit from sleep (8–15). For instance, if one tries to reach a target on a computer screen with the mouse while the cursor is rotated systematically without the subject noticing, the brain slowly learns to compensate for the rotation (16). Remarkably, sleep after learning results in movements that become more accurate and less noisy, in line with an increase in signal-to-noise ratio (S/N) (11). Sleep may exert similar effects on declarative memories by helping to extract the “gist” out of a large number of memories (5, 17): the details (noise) are weeded out, while the general idea (signal) is preserved. Sleep can also benefit the integration of new with old memories (5, 17), perhaps through similar means. During sleep, a large number of circuits can be activated off-line in many different combinations, without worrying about the consequences for behavior. In this way, new memories that fit better with the vast amount of organized older memories can be preserved, whereas other synaptic traces can be eliminated. So far, it seems that the memory benefits of sleep, especially for declarative memories, are due primarily to NREM sleep, but in some instances REM sleep or a combination of NREM-REM cycles may also play a role.

How does sleep produce its beneficial effects on memory? A commonly held view is that sleep may work by “reactivating” memories off-line and further strengthening them, or by transferring memories from short-lasting stores, such as the hippocampus, to long-term stores, such as the cerebral cortex, through a process akin to long-term potentiation of synapses (2, 7, 18). This view is an inference stemming largely from juxtaposing two lines of evidence. First, there is substantial evidence that activity patterns learned in wake are reactivated, or “replayed,” during sleep [e.g., Ref. (19–24)], suggesting that sleep may offer an opportunity for off-line memory rehearsal and, potentially, for synaptic strengthening of the kind observed during learning in wake. Second, the evidence for sleep-dependent memory consolidation mentioned above is taken as an indication that sleep may literally “strengthen” memories. Indeed, recent experiments have shown that increasing the intensity of neural activity during sleep inspecific circuits can benefit certain memories, consistent with an active role of sleep-dependent reactivation for memory consolidation (25, 26).

There are, however, alternative possibilities. It is now clear that the spontaneous activation of neural circuits embodying memories occurs not just during sleep after learning, but also in wake (27–31), as well as before learning (32). Moreover, most, if not all examples of consolidation of declarative memories after sleep do not reflect an absolute improvement, but rather a reduction in forgetting. Also, most examples of gist extraction and memory integration (33–35), are not consistent with the idea that sleep leads to the formation of new associations, but rather to the unmasking of ones that were already available (34, 36, 37). Finally, if new associations could easily be formed during sleep with mechanisms similar to those employed by the brain during wake, the brain would reinforce modes of functioning that become progressively divorced from the environment, potentially forming maladaptive “fantasies.”

An alternative scenario that can account for activity-dependent memory benefits of sleep, without incurring such problems, is that sleep may enforce a competitive *down-selection* of synapses, rather than a further strengthening or induction of new associative links. This *synaptic homeostasis hypothesis* (38–40) predicts that the competitive, activity-dependent depression of synapses can lead to memory consolidation due to an increase in S/N in both procedural and declarative tasks. Moreover, the same mechanism can give rise to gist extraction and the integration of old with new memories. In this way, off-line net synaptic depression during sleep can address the plasticity-stability dilemma (42, 43) in a satisfactory manner. Also, synaptic down-selection during sleep can restore the selectivity of neuronal responses (44), desaturate the brain’s ability to learn (45, 46), and reestablish cellular homeostasis challenged by net synaptic strengthening during wake (38–40).

In what follows, we show with computer simulations of simple, representative examples that a competitive, activity-dependent mechanism of synaptic down-selection during sleep can indeed account, at least in principle, for many of the benefits of sleep on memory. In a companion paper (41), we argue that biasing neural plasticity toward synaptic potentiation in wake and depression in sleep ensures, over repeated cycles of wake-sleep, that neural circuits learn to match the statistical structure of the environment without becoming prone to catastrophic interference or to spurious modes of functioning.

## 2 Materials and Methods

The goal of this paper is to demonstrate how, in principle, activity-dependent synaptic down-selection during sleep can account for the benefits of sleep under a number of different learning and memory paradigms. For this purpose, we make a number of simplifying assumptions and resort to networks of integrate and fire neurons based on previous work (47). These simulated networks fall in-between the large-scale, detailed Hodgkin–Huxley conductance-based models we previously employed to assess the effects of sleep on procedural learning (48) and the schema-level abstract models used by Lewis et al. (17) to discuss gist formation and memory integration during sleep. Below, we describe the neuron model, the plasticity rules employed for both training in wake and down-selection in sleep, and the spontaneous activation of stored memories in sleep. A limitation of this study is that different simulated networks were used for different experiments in order to most economically address different aspects of memory (procedural, declarative, hierarchical, and so on). However, it must be emphasized that all experiments utilize the same neuron and synapse model. Furthermore, the learning rule synaptic potentiation in wake paired with synaptic down-selection in sleep was also kept constant across the different experiments.

### 2.1 Neuron model

As in Ref. (47), we model integrate and fire neurons. Neurons communicate through voltage-independent feedforward connections as well as through voltage-dependent longer timescale feedback connections (49). Each neuron also receives a number of “external noise” connections that are assumed to originate outside of the network and are voltage-independent. Neurons evaluate on a 1 ms time step, in which the membrane potential V of a neuron is updated using the rules described below:

Here, *M*, *N*, and *O* are the number of feedforward, feedback, and external noise connections respectively. For each time step, the membrane potential is evaluated as follows: first, the contributions to the membrane potential from the voltage-independent connections (that is, due to feedforward and external noise) are evaluated; next, if the value of exceeds a pre-specified voltage-dependent depolarization threshold *Z*th, the contribution of the feedback connections is evaluated and is added to the final membrane potential *V* of the neuron. To reflect the longer timescale effects of NMDA-dominated feedback connections (49), feedback activity is integrated over the past two time steps, as indicated by equation (2) (results were qualitatively similar when integrating over 3–10 time steps, due to the short feedforward-feedback loops in the small networks employed in the present simulations). Once the final membrane potential *V* of the neuron has been evaluated, the output activity *A* of the neuron is evaluated using the following rule:

(1) $$V=\left\{\begin{array}{c} V_{PreFB},\;V_{PreFB}<Z_{th}\;\\ V_{PreFB}+V_{FB},\;\text{otherwise}\;\\ \; \end{array}\right.$$

(2) $$V_{FB}=\sum_{i=1}^{N}\;W_{i}^{FB}A_{i}^{FB}\left(t-1\right)+\sum_{i=1}^{N}\;W_{i}^{FB}A_{i}^{FB}\left(t-2\right)$$

(3) $$V_{PreFB}=V_{FF}+V_{\text{Noise}}$$

(4) $$V_{FF}=\sum_{j=1}^{M}\;W_{j}^{FF}A_{j}^{FF}\left(t-1\right)$$

(5) $$V_{\text{Noise}}=\sum_{k=1}^{O}\;W_{k}^{\text{Noise}}A_{k}^{\text{Noise}}\left(t\right)$$

(6) $$A\left(t\right)=\left\{\begin{array}{c} 1,\;V>\text{threshold}\;\\ 0,\;\text{otherwise}\; \end{array}\right.$$

Finally, at the end of every simulation time step, whether a neuron bursts or not, its membrane potential is reset to 0. This means that our simple spiking neuron model does not retain temporal history of the activity between time steps. This design point was chosen to minimize the complexity of the simulated examples.

### 2.2 Plasticity mechanisms: Synaptic potentiation in wake

In the present simulations, building upon previous work (47), we assume that synaptic potentiation in wake is dependent on four conditions: the neuron receives strong feedforward firing (bursts), the neuron receives strong feedback bursts, the neuron itself bursts strongly, and global neuromodulators are high (50, 51). These conditions capture some simple heuristics that neurons should follow when deciding which synapses to potentiate during learning. First, a neuron should be particularly sensitive to “suspicious coincidences” in input firing, both in time and in space. The reason is that, under the conditions of sparse firing mandated by energy constraints, such suspicious coincidences reflect the occurrence of events that happen more frequently than expected by chance, and which are thus ultimately related to the causal structure of the environment (52). Moreover, a neuron should be especially sensitive to coincidences between feedforward and feedback signaling, the former relayed by driving, primarily AMPA connections, the latter by modulatory, primarily NMDA connections (49). The reason is that coincidence between feedforward and feedback inputs suggests that the firing of the neuron has played a causal role in closing the loop in a neural circuit (41). It also indicates that the feedforward suspicious coincidences the neuron has captured, presumably originating in the environment, can be matched internally by feedback coincidences generated higher-up in the brain. This is a sign that the brain can internally model the suspicious coincidences it captures externally and vice-versa – a good recipe for increasing the matching between its causal structure and that of the environment (40). In addition, a neuron should pay particular attention if there is a positive correlation between presynaptic and postsynaptic spikes, as this suggests that there were enough suspicious coincidences, integrating over its many inputs, to make it fire within a restricted time frame (tens to hundreds of milliseconds). Finally, a neuron should only enable the strengthening of connections when it is awake and engaged in situations worth remembering, as signaled globally by neuromodulatory systems that are active during wake and especially during salient, unexpected, or rewarding circumstances, such as norepinephrine (53).

**Figure 1:**
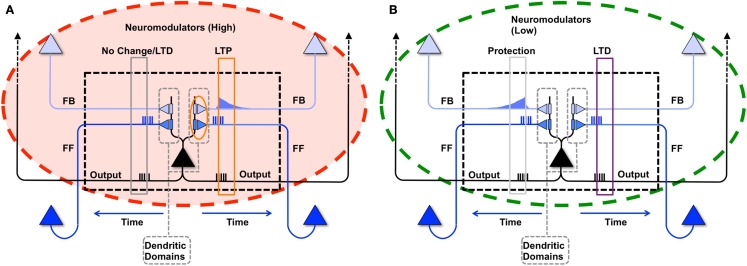
**Synaptic potentiation in wake and down-selection in sleep**. **(A)** During wake, plasticity is dominated by potentiation. Synapses are potentiated when a neuron receives persistent feedforward activation and longer timescale feedback activations on the same dendritic domain, the neuron itself exhibits strong activation, and global neuromodulators are present. The orange box indicates the dendritic domain which meets these requirements for LTP. Conversely, the gray box indicates a dendritic domain which is missing feedback activity, so either no change happens or LTD is induced. **(B)** In sleep, global neuromodulators are largely absent. The synapses in a dendritic domain are protected when the neuron is strongly activated by matching feedforward and feedback activations (gray box, left dendritic domain). Conversely, LTD occurs when a neuron bursts but its feedforward and feedback are mismatched within a dendritic domain (purple box, right dendritic domain).

This learning rule is graphically depicted in Figure 1A. Note that both the voltage-independent feedforward connections and the voltage-dependent feedback connections are assumed to be plastic. This figure also demonstrates that synaptic potentiating events are confined within a *dendritic domain* – that is, potentiation occurs if the aforementioned feedforward and feedback activations are matched on the same dendritic branch, in line with recent evidence (54, 55).

In the simulations studied in this paper, a dendritic domain is composed of at least one voltage-independent feedforward connection and one voltage-dependent feedback connection having a longer timescale. Therefore, this simple integrate and fire model captures both the inter-neuronal competition (between neurons) as well as the intra-neuronal competition (between the dendritic domains of a single neuron). If a neuron bursts, but receives feedforward and feedback on different dendritic domains, no potentiation is induced. In the present simulations, there is no long-term depression in wake, although in more realistic scenarios, some degree of synaptic depression is expected to occur [see Ref. (40, 48)].

As defined above, our simple dendritic domain model has at least one feedforward and one feedback connection, though several of the experiments described below employ multiple feedforward and feedback connections impinging upon a single dendritic domain. Furthermore, as proposed by the synaptic homeostasis hypothesis and captured by the learning rule described above, major synaptic changes require an interaction between feedforward and feedback connectivity. A more realistic model would account for the true spatial distributions of the modeled synapses and time-varying conductances; however, in this model, we capture the simple interactions of “proximal” feedforward and feedback synapses by organizing them into a dendritic domain.

Formally, if a neuron bursts because it received bursts on its feedforward and feedback synapses within the same dendritic domain, then the weight corresponding to a synapse *i* within the active dendritic domain is potentiated using the following rule:

(7) $$W_{i}=\left\{\begin{array}{c} W_{i}+\alpha ,\;\text{if }A_{i}\left(t-1\right)=1\text{ and }A_{\text{Domain}}\left(t-1\right)>0\;\\ W_{i},\;\text{otherwise}\;\\ \; \end{array}\right.$$

(8) $$A_{\text{Domain}}\left(t-1\right)=\sum_{m=1}^{M}\;A_{m}^{FF}\left(t-1\right)\sum_{n=1}^{N}\;A_{n}^{FB}\left(t-1\right)$$

That is, if a neuron bursts, then for each of its dendritic domains that receive both feedforward and feedback bursts, the active synapses are incremented by α. M and N are the number of feedforward and feedback synapses within the domain respectively. For the present simulations, the value of α is set to 0.1 for feedforward and 0.05 for feedback connections. Furthermore, feedforward and feedback connections are bounded between 0 and *W*_max_. For simplicity, global neuromodulators are assumed to be high throughout learning in wake, resulting in net potentiation, in line with experimental results (50, 51).

### 2.3 Plasticity mechanisms: Synaptic down-selection in sleep

The synaptic homeostasis hypothesis suggests that the net synaptic potentiation of wake must be balanced by synaptic depression in sleep. Various synaptic rules enforcing depression during sleep can be envisioned, including a proportional scaling down of all synapses (16), a rule biasing depression to spare stronger synapses more than weaker ones (48), and a down-selection rule – essentially a mirror image of the activity-dependent rule for synaptic potentiation in wake – implemented in the present simulations. As illustrated schematically in Figure 1B, a neuron that detects suspicious coincidences “protects” the associated synapses from depression during sleep, rather than further potentiating them. Possible cellular mechanisms include (1) the blockade of calcineurin, whose expression is upregulated in sleep (56) and promotes synaptic depression (56– 59), by high Ca^++^ levels; and (2) the selective entry of Arc in the spines that are not protected (60, 61). By contrast, synapses that are activated in isolation are not protected and thus depress progressively in the course of sleep. The switch to the down-selection mode is signaled globally by a drop in the level of neuromodulators (50, 51).

As with the potentiation mechanisms for wake described above, down-selection in sleep is assumed to be confined to dendritic domains as well. Formally, when neuron bursts during sleep, but receives either only feedforward bursts or only feedback bursts on synapses within a dendritic domain, it depresses a synapse *i* in a non-active dendritic domain according to the following rule:

(9) $$\begin{array}{l} W_{i}=\left\{\begin{array}{c} W_{i}-\beta ,\;\text{if }A_{i}\left(t-1\right)=1\;\text{and}\;A_{\text{Domain}}\left(t-1\right)=0\;\\ W_{i},\;\text{otherwise}\;\\ \; \end{array}\right. \end{array}$$

That is, if a neuron bursts and has either received feedforward without feedback or feedback without feedforward bursts on synapses within a dendritic domain, the active synapses in that domain are depressed by β. For our simulations, the value of β is set to be 0.01 for feedforward and 0.005 for feedback connections. In the experiments described in this manuscript, the values of α and β were empirically chosen based on our simulation’s constraints, including the degree of connectivity, average input of the neuron, and simulation time. Feedback rates were chosen to be smaller than feedforward rates such that top-down connections would change more slowly, while feedforward connections could change more rapidly with new input activity from the environment.

Alternative down-selection rules were investigated in pilot simulations. One such rule prescribed that, when a neuron does not burst, synapses in active dendritic domains are depressed, in line with classic Hebbian and spike-timing-dependent plasticity (STDP) learning rules (62–64). Qualitatively, the results for the simple simulations presented in this paper were not substantially different from those obtained by employing the rule in Figure 1B.

### 2.4 Initial connectivity, neuronal groups, and spontaneous activation in sleep

In the simulations, neurons are arranged in highly connected neuronal groups (typically 18 integrate and fire neurons) with feedforward and feedback connections, in line with the minicolumn organization of sensory cortices (65). Within our simplified models, highly interconnected neuronal groups ensure that activity levels are stable, and that inter-group connectivity, which is the focus of our analysis, is not too sparse.

The number of neurons and their overall connectivity varies across the different experiments and will be described in the later sections. In all simulations, the synaptic weights corresponding to feedforward and feedback connections are initialized to 1/10 of their maximum value (*W*_max_/10), with no initial preference to any input patterns. Each of the neurons also receives three noise connections, initialized to a value of *W*_max_, which are not plastic. The threshold of the neurons is set to 2*W*_max_, such that two inputs of maximum strength are sufficient to make the neuron fire. Simultaneous activations of 2 or more noise inputs result in a postsynaptic neuron burst, allowing the neurons to become spontaneously active even before synapses have strengthened. Finally, as mentioned above, the voltage-dependent feedback connections are integrated if the neuron is depolarized beyond the value of *Z*_th_, set here to *W*_max_/5 (from either noise inputs or voltage-independent feedforward connections).

During simulated slow wave sleep, neurons undergo slow oscillations at around 1 Hz, during which they alternate between depolarized up-states when they fire in a way similar to wake, and hyperpolarized down-states, during which they tend to remain silent. To provide a simple implementation of such slow oscillations, the level of activation of noise connections was set to 20% for a total of 500 ms (up-state), and to 5% for the next 500 ms (down-state). Neurons become spontaneously activated, and through spike percolation in the subsequent time steps, activate memories through the network. When connections are strong, as is often the case after forming a new memory, a few spontaneous spikes may be sufficient to reactivate an entire memory over a few time steps.

## 3 Results

### 3.1 Consolidation of procedural memories

Procedural (or non-declarative) memories are those that do not require conscious control or awareness. In the following section, we investigate procedural learning under the plasticity and down-selection mechanisms outlined above and verify their effects using computer simulations.

#### 3.1.1 Procedural learning experiment

**Figure 2:**
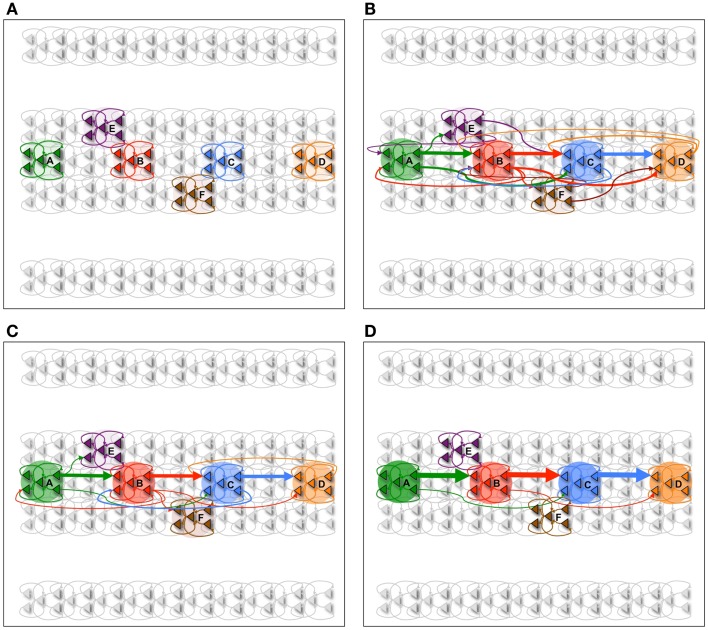
**Procedural learning task**. **(A)** The network is initialized to have weak connections between all neural groups. **(B)** During wake, the sequence A ⇒ B ⇒ C ⇒ D is learned. However, other connections are also potentiated due to spontaneous activations as well as mistake sequences encountered during learning. Neuronal group color saturation indicates its frequency of activation in wake. **(C)** After sleep, activation protects the learned sequence significantly more than the other connections between groups, and the sharpness of selectivity is restored and the S/N is enhanced. Neuronal group color saturation indicates its frequency of activation in sleep. **(D)** After several training and renormalization periods, the only significant connections are the ones that capture the learned sequence. Neuronal group color saturation indicates its frequency of activation in sleep. Connection strengths less that 10% of the maximum are not shown for simplicity.

In Figure 2, we consider a neuronal network learning a procedural task. Six neuronal groups (composed of 18 neurons each) are shown and labeled in Figure 2A. The procedural memory task is modeled in such a way that learning happens in a segregated channel, where activity percolates from left to right. To place this experiment within the context of a real-world example, the sequential task can be compared to learning a key progression on a piano.

The network is trained on the sequence A ⇒ B ⇒ C ⇒ D in pair-wise steps. First, the network is repeatedly exposed to the sequence A ⇒ B, then to the sequence B ⇒ C, and finally to the sequence C ⇒ D. This simulates the human behavior where a complex sequence is learned by decomposing it into simple sub-sequences. The network is trained on the correct sequence 90% of the time, while 10% of the time the network is trained on one of two “spurious” sequences (A ⇒ E ⇒ C ⇒ D and A ⇒ B ⇒ F ⇒ D). These erroneous sequences correspond to mistakes (i.e., noise) that may be made when learning a new scale on the piano.

**Figure 3:**
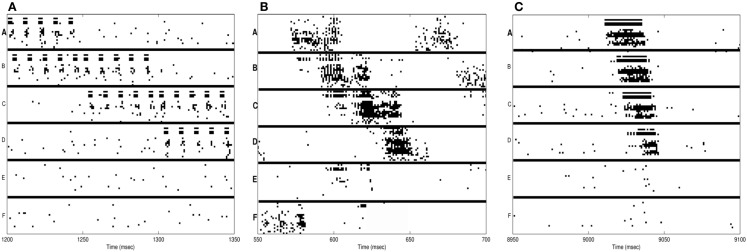
**Neuron activations during procedural learning task**. **(A)** Spike raster plot of the training phase. Stimuli A and B are followed by the stimuli B and C, which are followed by C and D. **(B)** Spike raster plot showing the activation of the correct sequence during sleep. **(C)** Spike raster plot showing the activation of the correct sequence after a single session of wake-training followed by renormalization in sleep.

In Figure 3A, we see the spike raster plots of the six neuronal groups during training. From the Figure, we see that the network is trained with A ⇒ B, B ⇒ C, and finally C ⇒ D. Figure 2B shows the changes in connection strength among the neural groups after training in wake. In the figure, the connection color corresponds to the source neuronal group, and the connection width corresponds to synaptic strength. One can see that the strongest connections (A ⇒ B ⇒ C ⇒ D) correspond to the learned sequence. However, other connections have also been strengthened, including those corresponding to “spurious” sequences.

As plasticity leads predominantly to potentiation during wake, the influence of the activation of several neuronal groups has broadened. For example, ideally neuronal group B would have a strong set of synapses to group C. However, due to spurious associations, B has established an effect on three additional neuronal groups. In such a setup, uninterrupted wake can lead to runaway potentiation, resulting eventually in strong connections between all neuronal groups and complete loss of specificity.

Because the neuronal groups corresponding to the signal (A ⇒ B ⇒ C ⇒ D) form stronger inter-group connections as compared to the groups involved only in spurious sequences (groups E and F), they are reactivated at a higher rate in sleep. As a result, the synapses corresponding to the correct procedural memory (A ⇒ B ⇒ C ⇒ D) are protected from depression, whereas the connections corresponding to spurious sequences are not. As renormalization mechanisms during sleep down-select the non-protected synapses, selectivity is enhanced – the connections underlying A ⇒ B ⇒ C ⇒ D remain strong after sleep, while many other connections are depressed. Figure 2C shows the connections strengths between various neuronal groups after a single period of wake-training and sleep. Finally, Figure 2D shows that after many training and sleep sessions, this effect is more pronounced.

#### 3.1.2 Sequence recall and S/N after procedural learning in wake

After learning in wake, performance and S/N are assessed by evaluating the networks ability to correctly recall the learned sequence in wake. To do so, neuronal group A is clamped in the activated state for 25 ms, while the ensuing activations of other neuronal groups are recorded. A correct recall is counted whenever at least 50% of the neurons in the groups B, C, and D are activated in the correct sequence A ⇒ B ⇒ C ⇒ D within 500 ms of clamping A in the activated state. If 50% of either neuronal group E or F are activated within this time window after stimulus onset, the recall is counted as incorrect. Performance is quantified as the percentage of correct recalls, while S/N is measured as:

(10) $$S∕N=\frac{\text{Number}\;\text{Correct}\;\text{Recalled}\;\text{Sequences}}{\text{Number}\;\text{Incorrect}\;\text{Recalled}\;\text{Sequences}}$$

Recall percentages and S/N values for the experiments described in the next subsections are summarized in Table 1.

**Table 1:** **Sequence recall performance and S/N for procedural learning**.

Experiment	Baseline – 0 wake/sleep cycles	1 Wake (before sleep)	1 Wake/sleep cycle	10 Wake/sleep cycles	Extra wake	Potentiation in sleep
Recall (%)	0	80	90	95	20	25
S/N	0	4	9	19	0.25	0.33

#### 3.1.3 Sequence reactivation during sleep

Neurons within the same group tend to be active at the same time due to the high degree of intra-group connectivity. As shown in Figure 3B, this activity often propagates between groups, “replaying” the newly learned sequence A ⇒ B ⇒ C ⇒ D. During simulated sleep periods, spontaneous activation of this kind leads to plastic changes mediating synaptic down-selection as described in Section 6. Accordingly, synapses are depressed in a manner inversely proportional to their reactivation frequency, leading to the connectivity shown in Figure 2D.

#### 3.1.4 Synaptic down-selection during sleep improves performance and S/N

The baseline network shown in Figure 2A (before training) achieves, as expected, a recall performance of 0% and S/N of 0 (Table 1). Next, the network is trained with the signal (A ⇒ B ⇒ C ⇒ D) and spurious sequences (A ⇒ E ⇒ C ⇒ D and A ⇒ B ⇒ F ⇒ D) as described in Section 1, for a total of 10,000 ms iterations. Immediately after this wake-training period, the network is able to recall the sequence correctly 80% of the time, resulting in a S/N of 4. The corresponding connectivity is shown in Figure 2B.

Subsequently, the network is allowed to undergo synaptic down-selection in the sleep mode for 10,000 ms. The resulting synaptic connectivity is shown in Figure 2C. After sleep, the recall performance of the network improved to 90% and the S/N measure increases to 9 (Table 1). Figure 3C shows a correct recall sequence of the network, after the network had experienced a single wake-sleep cycle.

Finally, the network undergoes 10 wake/sleep cycles. As a result, the synapses corresponding to the correct sequence are significantly strengthened, whereas spurious connections have been down-selected, as shown in Figure 2D. The recall rate is 95% and the S/N measure increased further to a value of 19.

#### 3.1.5 Extra wake degrades performance and S/N

**Figure 4:**
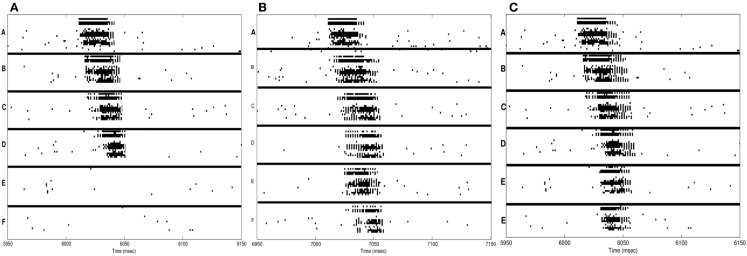
**Sequence recall**. **(A)** With sleep between training and testing sessions, the spike raster plot shows that the correct sequence is recalled correctly after a single wake-sleep cycle. **(B)** After two training sessions without sleep, the spike raster plot shows the activation of the incorrect sequence by activating the neural group A. **(C)** After synaptic potentiation during sleep, the spike raster plot shows the activation of the incorrect sequence.

In Figure 4A, the spike-raster plot shows the correct recall of the sequence after a single period of wake followed by sleep-dependent down-selection. To establish whether synaptic down-selection in sleep can be substituted for by additional learning in wake, we simulate an additional wake-training period without intervening sleep. Accordingly, the network is trained in the wake mode for 20,000 ms. Figure 4B shows the spike raster plot of a typical recall sequence after the extended training period. Since the connections to the neuronal groups associated with the spurious sequences (E and F) have been further potentiated (while those associated with the correct sequences were already saturated), the correct sequence is recalled only 20% of the time, corresponding to an S/N measure of 0.25 (Table 1).

#### 3.1.6 Synaptic potentiation during sleep degrades performance and S/N

Next, we investigate the consequences of permitting synaptic potentiation, rather than down-selection, to occur during the slow oscillations occurring in sleep. For this purpose, the learning rule in the sleep mode is switched from the down-selection rule (Section 3) to the potentiation rule normally associated with wake (Section 2). An example of the networks recall performance is shown in the raster plot of Figure 4C. This example is representative of the fact that, with potentiation occurring during sleep, the neuronal groups involved in the spurious sequences (groups E and F) are activated quite frequently. As a result, the correct sequence was recalled only 25% of the time, corresponding to a S/N value of 0.33.

### 3.2 Consolidation of declarative memories

**Figure 5:**
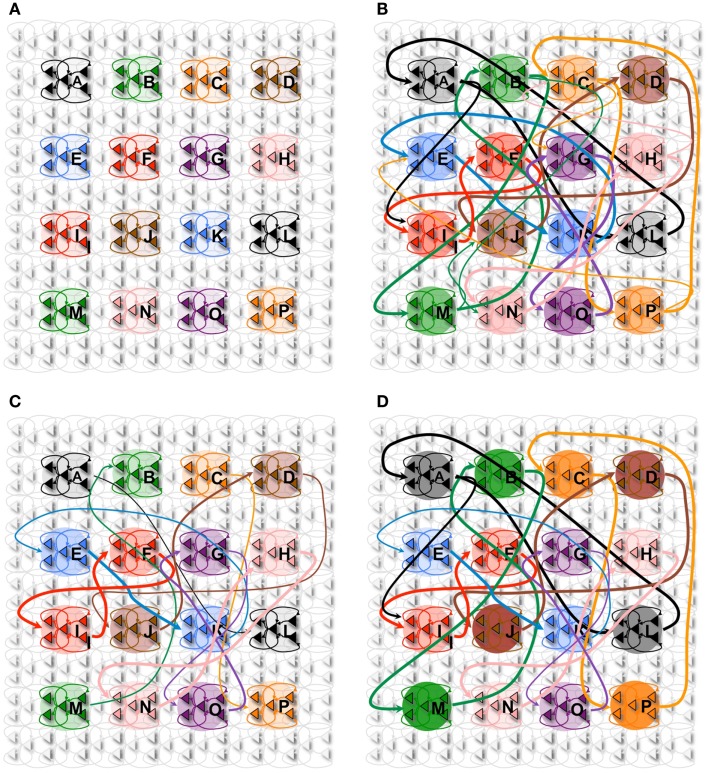
**Declarative memory task**. **(A)** The network with 16 neuronal groups before it is trained. **(B)** The network with 16 neuronal groups is trained to learn 8 pair associations. However, other connections are also potentiated due to spontaneous activations as well as mistake sequences encountered during learning. Neuronal group color saturation indicates its frequency of activation in wake. **(C)** After sleep, activation protects the learned pairs significantly more than the other connections between groups. Neuronal group color saturation indicates its frequency of activation in sleep. **(D)** After training in wake (B), half of the associative pairs are cued during sleep (Pairs A-L, B-M, C-P, D-J). The neuronal group color saturation indicates its frequency of activation in sleep, which is greater for the cued pairs. As a result, cued pairs are significantly protected and subsequently have a higher recall rate and S/N.

In this section, we simulate the effects of synaptic down-selection during sleep on conscious, declarative memories. To keep the model as simple as possible, we do not attempt to simulate explicitly a hippocampal-neocortical network, which would go beyond the scope of the present paper. Rather, we make use of a network connected as an associative matrix with inter-group connections between all neuronal groups (see Figure 5), instead of within a segregated channel, as was done in the previous section to simulate procedural learning. The encoding of arbitrary associations is a classic paradigm employed in studies of declarative memory consolidation (66, 67).

#### 3.2.1 Declarative learning experiment

In Figure 5A, we consider a network composed of 16 neuronal groups. The neuronal groups are configured to identify one of 16 different patterns, A through P. During training in wake, pairs of neuronal groups are simultaneously activated. Each of the pairs is activated for 2,000 ms to induce strong synaptic potentiation.

Figure 5B shows the connectivity established among the neuronal groups once the network has been trained with 8 pairs of associations (e.g., A and L, B and M). In this experiment, we stimulate one neuronal group and observed whether 50% (or more) of the neurons in its associated neuronal group fired within 50 ms of the onset of stimulation. For example, we stimulate neuronal group A and observe whether neuronal group L is correctly recalled (activated). As with the procedural memory task, if any neuronal group outside of the paired associate showed at least 50% activation within this window, the trial is counted as an incorrect recall. The S/N for the declarative memory task is defined as:

(11) $$\text{S/N}=\frac{\text{Percentage}\;\text{Correct}\;\text{Recalled}\;\text{Pairs}}{\text{Percentage}\;\text{Incorrect}\;\text{Recalled}\;\text{Pairs}}$$

#### 3.2.2 Synaptic down-selection during sleep preserves performance and S/N

Initially, the network shown in Figure 5A has no strong associative links, the baseline recall rate is 0%, and the S/N is 0. Immediately after the training phase, the strengthening of connections demonstrated in Figure 5B leads to a recall rate of 87%, corresponding to a S/N of 6.7 (see Table 2). After a period of sleep (see Figure 5C), the network recalls the learned pairs correctly 75% of the time, with a S/N of 2.7. If the network is allowed to undergo 10 wake/sleep cycles, the recall rate improves to 94%, with a S/N of 15.6.

**Table 2:** **Paired associate recall performance and S/N for declarative learning**.

Experiment	Baseline – 0 wake/sleep cycles	1 Wake (before sleep)	1 Wake/sleep cycle	10 Wake/sleep cycles	Extra wake	Potentiation in sleep	Cue in sleep
Recall (%)	0	87	75	94	53	60	85
S/N	0	6.7	3	15.6	1.12	1.5	5.67

#### 3.2.3 Extra wake degrades performance and S/N

Next, we simulate the standard experimental comparison between the effects of sleep after training versus extra wake. Of the two identical networks trained on the associative recall task, one is allowed to enter the sleep mode for 20,000 ms (as described above), while the other is kept in the wake mode for the same amount of time. To mimic interference due to spurious associations occurring in wakefulness, the second network is exposed to six random associations, different from the ones learned during the initial training. Each of these spurious associations is presented to the network for 300 ms, leading to further synaptic potentiation. As a result, the awake network shows correct recall only 53% of the time, corresponding to a S/N value of 1.12.

The results obtained after declarative learning should be compared to the one obtained with procedural learning above. With the procedural task, recall after training in wake is 80% and after down-selection in sleep it improves to 90%. With the declarative task, recall after training in wake is 87% and after down-selection in sleep it is reduced to 75%, compared to 53% without sleep. This difference between absolute improvement after procedural learning and relative preservation (reduced degradation) after declarative learning is in line with experimental results (2). In the simulations, these results can be explained by considering that, in the procedural task, acquisition of both correct and spurious associations through synaptic potentiation in wake is confined within a single channel, reducing the effects of interference. By contrast, in the declarative task, the all-to-all connectivity that is characteristic of associative matrices is much more prone to interference by spurious associations.

#### 3.2.4 Synaptic potentiation during sleep degrades performance and S/N

Next, we examine the effects of synaptic potentiation during sleep. For this experiment, the network is trained as before with 8 associated pairs for 2,000 ms. However, instead of synaptic down-selection, the network uses the potentiation rule as in wake (see Section 2.2). As a consequence, the recall rate of the network degrades to 60%, with a S/N value of 1.5. Examining the dynamics of activation and plasticity during sleep revealed that the primary reason for this degradation in performance and S/N is the spurious strengthening of connections between neuronal groups that are spontaneously activated during sleep.

#### 3.2.5 Cuing memories during sleep improves performance and S/N

Finally, based on recent experimental results [e.g., Ref. (68, 69)], we also examine the effects of stimulus cuing during sleep. As before, the network shown in Figure 5B is trained in wake to learn 8 associative pairs. After training, the network has a recall rate of 87% and a S/N measure of 6.7. During sleep, 4 of the trained pairs are cued (i.e., strongly activated through external stimuli), while the other 4 pairs are activated as before only through the occurrence of spontaneous slow oscillations. The external cues are presented to the network 3 times each (1 pair at a time) for a period of 500 ms per cue. The total sleep time was again 20,000 ms.

During external cuing in sleep, the plasticity of the network was still governed by synaptic down-selection. Each of the cued neuronal groups spent on average 8% more time in the up-state during slow wave oscillations. Because each of the cued pairs were activated together as a result of the stimulation, the synapses between them were better protected than those between the non-cued pairs, which became activated only through the simulated slow wave oscillations.

Figure 5D shows the state of the network after the cuing in sleep. The color saturation of each neuronal group indicates its intensity of activation during sleep, which has a direct impact on the synaptic strength between the associated pairs. When we measure recall performance for the entire network after 1 sleep cycle, the value is 85%, corresponding to a S/N value of 5.7, higher than without cuing. We then compare directly the cued with the non-cued pairs. As expected, the non-cued pairs have a recall rate of 75% with an S/N of 3, values similar to those observed without cuing (see Section 2). By contrast, the cued pairs have a recall rate of 95% and a S/N measure of 19, an improvement in line with empirical results (68, 69).

Alternatively, if synaptic potentiation occurs during sleep, the network again runs the risk of strengthening spurious connections, as described in Section 4. If potentiation is limited only to cued pairs, while non-cued pairs undergo synaptic down-selection, the results are again in line with those shown in Figure 5D; however, such a result implies that during sleep the brain would have a way to implement different plasticity rules for cued and non-cued pairs and to differentially tag the relevant synapses during wakefulness.

### 3.3 Gist extraction

Recent evidence indicates that sleep facilitates gist extraction – the ability to form enduring memories of high-level invariants, such as faces, places, or even maps, more than of low-level details (5, 17). Below, we examine how spontaneous activation followed by synaptic down-selection in sleep can promote gist extraction in a hierarchically organized network.

#### 3.3.1 Synaptic down-selection during sleep favors gist extraction in hierarchically organized networks

**Figure 6:**
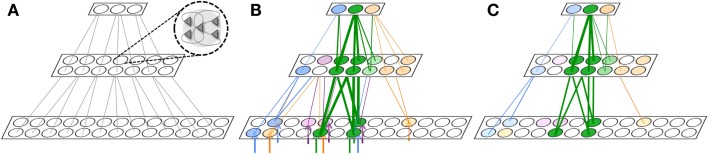
**Gist extraction task**. **(A)** The hierarchically organized neuronal network is initialized to with weak connections shown in gray. **(B)** During wake, the network is exposed to four stimuli. The blue, purple, and orange stimuli partially overlap with the green stimuli. After learning, many connections are strengthened, though the connections corresponding to the overlapping features in the stimuli are strongest. Neuronal group color saturation indicates its frequency of activation in wake. **(C)** During renormalization, many connections are depressed, but the gist remains. Neuronal group color saturation indicates its frequency of activation in sleep. For **(B,C)**, connection strengths less than 10% of the maximum weight are not shown for simplicity.

For this purpose, we arrange neuronal groups according to the simple hierarchical organization shown in Figure 6. This network architecture is inspired by the hierarchical organization of anatomical connections in the cerebral cortex and by the physiological evidence showing that neurons in higher regions respond to more invariant stimuli than neurons in lower regions (70). In Figure 6A, each circle represents a neuronal group of 18 interconnected neurons. For this experiment, the first level contains 26 neuronal groups, the middle level 14, and the upper level 3. To ensure that the top level of the network receives feedback activations necessary to trigger plasticity, it is recurrently connected to a fourth level via non-plastic synapses (not shown). As with other experiments, feedforward and feedback connections are initialized to *W*_max_/10 (though feedback connections are not shown to avoid clutter).

Figure 6B shows four different stimuli on which the network is trained during wake, indicated by the colored arrow inputs to the bottom level of the network. Note that the orange, purple, and blue stimuli each partially overlap with the green stimuli. During wake, each of the four stimuli is presented to the network for 500 ms, four times each, with the order chosen randomly. The total training time in wake is 8,000 ms.

Figure 6B shows the feedforward connections after training in wake, where the thickness of the connections corresponds to their synaptic weight. While many connections became stronger, those relaying features that overlapped among the different stimuli – that is, the gist – show the largest increase in strength (at or close to *W*_max_; connections with strength at or less than *W*_max_/10 are omitted for clarity).

In Figure 6C, the color intensity of each of the neuronal groups indicates its frequency of activation during a subsequent period of sleep lasting 10,000 ms. As also shown in the figure, due to down-selection during sleep, many of the synapses are depressed, but those corresponding to the gist shared by the four stimuli are mostly preserved. That is, during down-selection in sleep the connections between level 2 and 3 are comparatively better protected than those between level 1 and 2. Thus, spontaneous activation and down-selection during sleep favor gist extraction.

#### 3.3.2 In hierarchically organized convergent-divergent networks, top-down spontaneous activation predominates over bottom-up activation during sleep

In the model, an important factor promoting the relative preservation of gist versus detail has to do with the dynamics of spontaneous activation during sleep. The general hypothesis is that neuronal patterns in sleep are more frequently activated top-down (level 3 before 2 before 1) than bottom-up (level 1 before 2 before 3).

To validate this notion, we perform two experiments. Using hierarchical network of neurons similar to that shown in Figure 6A, feedforward and feedback weights are initialized to *W*_max_/5. Furthermore, the timescale of the feedback connections is set equal to the timescale of feedforward connections (1 ms), though feedback connections maintained their voltage-dependent behavior. For this experiment, while the network is engaged in spontaneous slow oscillations through noise injections, synaptic down-selection is turned off. A random neuron is chosen in level 1 and level 3, and we observe the frequency with which the neuron initiated a spike that will percolate through the hierarchy. Percolation is successful if, for example, the neuron in level 3 spikes, then in the next time step a neuron (within the first neuron’s receptive field) in level 2 also spikes, and finally a neuron (within the first neuron’s receptive field) spikes in level 1. Bottom-up sequences are detected the same way, but in the opposite direction (level 1 to 2 to 3). After 10,000 ms of simulated sleep, the number of top-down initiated sequences was nearly 4 times that of bottom-up sequences.

In our second experiment, we take the same network (in simulated slow wave sleep) and impose a steady activation of either 1/3 of the neurons in the top level (level 3), chosen at random, or of 1/3 of the neurons in the bottom level (level 1). For this experiment, the average firing rate of the network is 5% higher when the stimulation is provided to the top level, despite the fact that when the stimulation is provided at the bottom level, substantially more neurons are activated (see Figure 6). In the model, this bias for top-down activation can be explained by the narrower fan-out of feedforward versus feedback connections in a convergent-divergent hierarchical network. In the bottom-up, feedforward direction, it is unlikely that the randomly stimulated neurons fit the receptive field of a higher-level neuron to make it fire. This unlikelihood is multiplied when considering the percolation of activity in a feedforward direction over multiple levels. By contrast, since a level 3 neuron has a broad fan-out to lower levels, it can bias toward firing many compatible patterns of activation in lower levels, so activation is more likely to propagate in top-down direction.

The bias for top-down initiated activity is enhanced further by the longer timescale effects typically associated with top-down connections. By increasing the timescale of feedback connections to 2 ms, the activity of the network is 20% higher when the top level is activated. The bias is even larger if the total number of feedback connections is made to be greater than the number of feedforward connections, as has been suggested for corticothalamic versus thalamocortical connections (71, 72).

### 3.4 Integration of old with new memories

All previous experiments (procedural learning, declarative learning, and gist extraction) begin with networks treated as a *tabula rasa*, without strong connections corresponding to previous memories. In the following experiments, we consider a hierarchically organized network of neurons with previously stored memories. In particular, we consider how synaptic down-selection in sleep favors new memories that fit well with old memories, and how synaptic depression in wake can interfere with old memories.

#### 3.4.1 Synaptic down-selection during sleep favors the integration of new with old memories

**Figure 7:**
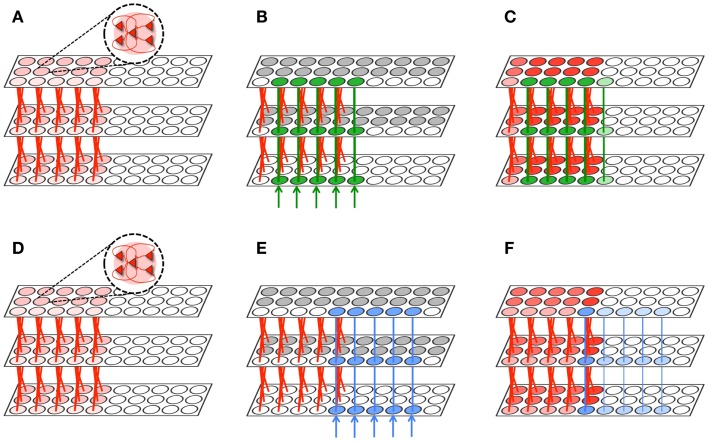
**Memory integration task**. **(A)** A hierarchically organized network contains a previously learned memory. **(B)** The first network is exposed to a stimulus which forms a new memory (green) that significantly overlaps with the old memory (red). Gray neuronal groups are inhibited. **(C)** During sleep, the green and red memories are often activated together. As a result, the memory is protected and downscaling is minimized (2% on average). Neuronal group color saturation indicates its frequency of activation. **(D)** The same initial network from **(A)**. **(E)** This network is exposed to a stimulus which forms a new memory (blue) that minimally overlaps with the old memory (red). Gray neuronal groups are inhibited. **(F)** Conversely, most of the blue memory is activated independently of the red memory. Connections are downscaled 28% between the bottom and middle level, and 11% between the middle and higher level. Neuronal group color saturation indicates its frequency of activation.

Both theoretical considerations (73) and experimental evidence (5, 17) suggest that off-line activation of neural circuits during sleep may provide an ideal setting for the integration of newly acquired associative links with an established body of knowledge within the brain. To assess how sleep-dependent down-selection mechanisms can aid this process of memory integration, we resort again to a hierarchically organized network of neuronal groups (Figure 7). Each of the 3 levels of the network comprised 30 neuronal groups containing 18 neurons each. As with the gist example, the top level of the network receives non-plastic feedback activations from a fourth level in order to initially trigger plasticity (not shown).

The two identical networks are then endowed with “old” memories (shown in red in Figures 7A,D, implemented by a set of strong feedforward and feedback connections between levels (initialized to *W*_max_). Then, both networks are exposed to a stimulus to create a “new” memory. The network in Figure 7B is exposed to a stimulus (shown in green) that has substantial overlap with the old red memories. Conversely, the network in Figure 7E is exposed to a stimulus (shown in blue) that only overlapped with a single neuronal group (per level) associated with the old red memories. To prevent extensive subliminal plasticity through the entire network of associations during wake, we keep the majority of the neuronal groups in the second and third levels of the network in an inactive state (shown in gray). In real brains, global activation and plasticity during experience is ruled out by several mechanisms of intra- and inter-areal competition and inhibition.

As shown in the figure, after a wake-training period of 10,000 ms, both networks has acquired the new memory by strengthening several connections between the levels (shown in blue and green, respectively). Thereafter, both networks are set in the sleep mode for an additional 10,000 ms. During simulated sleep, the new green memory (Figure 7C) is often co-activated with the old red memories, due to the presence of many points of contact. In the figure, the frequency of activation is indicated by the color saturation of the neuronal group. Since connections belonging to the new green and old red memories often terminate on the same synaptic compartments, even partial activations are able to protect synapses from down-selection, as long as activations are conveyed by both feedforward and feedback connections. As a result, the synaptic weights of the new green memory are depressed, on average, by only 2%. Conversely, a significant portion of the new blue memory (Figure 7F) can only be activated by the other neuronal groups within the blue memory. As a result, the blue memory often encountered mismatched feedforward and feedback activations, resulting in substantial depression: synapses between levels 1 and 2 are depressed on average by 28%, and those between level 2 and 3 by 11%. Thus, as expected, spontaneous activation in the sleep mode protected preferentially the new memory that overlapped significantly with old memories, resulting in memory integration.

#### 3.4.2 Synaptic depression in wake degrades old memories that are not activated

As mentioned in the Section “Introduction,” down-selection should occur during sleep, when the brain is off-line and it can comprehensively activate its memory bases. By contrast, if the necessary renormalization of synaptic strength are to happen in wake (concurrently with learning by potentiation), it will run the risk of degrading important memories simply because they happen not to be activated during the limited sampling dictated by a day’s wake behavior.

To illustrate this aspect with a simple example, in this final simulation we implement renormalization during wake while a new memory was being formed. The same network shown in Figure 7A is exposed to the green stimulus as in Figure 7B. As in the previous simulation, during wake-training the majority of the neuronal groups in the second and third levels of the network, corresponding to old memories, are kept in an inactive state (shown in gray). This is done to replicate the sampling bias occurring during any particular wake episode: on a given day, the brain is typically faced with some novel stimuli (relating, say, to a new acquaintance), and has no opportunity to activate most of its old memories (including memories related, say, to one’s old friends).

To achieve activity-dependent synaptic renormalization during wake, the plasticity rule is modified by allowing for the depression of synapses that are inactive when the postsynaptic neuron fired, not unlike STDP (62–64). During wake-training, neurons responding to the features of the new stimulus (related, say, to the face, voice, posture etc. of a new acquaintance) are activated often, so the active synapses conveying the new stimulus became progressively stronger, laying down a trace for the new memory (green in Figure 7B). However, on the same day, synapses onto the same neurons originating from neurons involved in old memories (related, say, to the face, voice, posture etc. of old friends) are almost always inactive, and undergo progressive depression (on average, by 90% when using the same learning rates for potentiation and depression). Thus, activity-dependent synaptic renormalization in wake will achieve new learning at the expense of potentially degrading important features of old memories. Clearly, it will not be advisable, when making a new friend, to run the risk that of losing the old ones simply because they were not present on that day.

During simulated sleep, the effects of the synaptic depression in wake are compounded. As the new memory no longer overlaps with the old memory, the advantages of co-activation in sleep are absent. As a result, the connections of the new memory are depressed to the same levels as they would have been were they independent of an old memory (similar to the network shown in Figure 7F.

## 4 Discussion

The goal of this paper is to show that cycles of activity-dependent synaptic potentiation during learning in wake, followed by down-selection of synapses during the slow oscillations of sleep, can account in principle for several of the beneficial effects of sleep on memory, including the consolidation of procedural and declarative memories, gist extraction, and the integration of new with old memories. These effects were demonstrated using computer simulations of simple integrate and fire neurons endowed with plasticity, which were mechanistic enough to evaluate whether the predicted effects could be achieved in a “neural” implementation, yet as simple as possible to focus on proof of principle rather than on detailed models of specific brain regions. Below, we discuss the rationale for the down-selection idea, some of the general mechanisms that could implement down-selection in the mammalian brain, the extent to which down-selection can account for the experimental results about sleep and memory, and some differences of the down-selection model with respect to the standard replay-transfer-potentiation model. In a companion paper (41), we examine how the process of learning during wake and down-selecting during sleep can be quantified in terms of how well a synaptic architecture captures and models the statistical structure of the environment.

### 4.1 Sleep and synaptic homeostasis

The sleep-dependent down-selection model investigated in this paper is motivated by the hypothesis that the core function of sleep is to renormalize synaptic strength, which increases during wake as a result of learning and during development as a result of synaptogenesis (38–40). A progressive increase in synaptic strength is not sustainable at the single neuron level due to the burden it poses on energy consumption, space and cellular supplies. Therefore, there needs to be an overall renormalization of synaptic strength to restore cellular and synaptic homeostasis. As hypothesized here, if synaptic renormalization happens through an activity-dependent, competitive down-selection process during sleep, several systems-level advantages can also be obtained, including memory consolidation, gist extraction, and integration of new with old memories, in addition to a desaturation of the ability to learn. The core prediction of a net increase in synaptic strength in wake and its net decrease in sleep is supported by molecular, electrophysiological, and structural evidence (38–40). Various memory benefits of sleep are also supported by much evidence (2–7). However, these results are often interpreted as consistent with the replay-transfer-potentiation model: (i) the reactivation of memories during sleep, especially declarative memories, leads to (ii) their transfer from hippocampus to cortex; (iii) a further potentiation of synapses that underlies their consolidation. As shown here, an alternative notion that spontaneous activity during sleep may actually produce a down-selection of synapses, the synaptic homeostasis hypothesis, is: (i) consistent with molecular, electrophysiological, and structural evidence; (ii) can provide a parsimonious account of many of the benefits of sleep on memory; (iii) avoids inherent risks associated with potentiating patterns of activity occurring when the brain is not on-line. Below, we briefly outline the rationale behind the synaptic homeostasis hypothesis and its prescriptions for wake and sleep.

### 4.2 Potentiation in wake

Neuronal firing, especially due to its postsynaptic consequences, is energetically more expensive than silence (74–78). Therefore, neurons should fire sparsely and do so only for important events (44). At any given time, a neuron deep in the brain can infer the importance of its inputs based on the occurrence of “suspicious coincidences” (52). Given that a neuron receives a large number of synapses, all of them usually firing at very low rates due to energy constraints, coincidences of firing at multiple synapses, occurring much above what would be expected by chance, are suspicious and probably important, as they reflect some statistical structure in the inputs and ultimately in the environment. A burst of spikes is also suspicious, suggesting the persistence of some input beyond chance (47). When a neuron detects the occurrence of suspicious coincidences by integrating over several of its synapses, in space or time, it should communicate that something presumably important happened by firing, rather than by silence; otherwise important events would not be able to percolate and be further integrated down-stream (44). In an open-ended environment, new contingencies occur all the time when one is awake, especially when exploring the environment, leading to new suspicious coincidences over synapses that may initially not be strong. In order to learn these new contingencies, a neuron must be able to potentiate these synapses so it can reliably signal their detection to the rest of the brain. In summary, *when connected to the environment, neurons should fire for suspicious coincidences and percolate them through the brain by potentiating the synapses that convey them.*

The learning rule implemented in the present model [Figure 1A, see also Ref. (47)] follows these prescriptions and, in line with those of Hebbian and spike-timing-dependent plasticity (79), requires the coincidence between pre- and postsynaptic firing. Moreover, it emphasizes suspicious coincidences in input firing that occur over a restricted dendritic domain (54, 55), especially if they are due to a coincidence between driving, feedforward (primarily AMPA) and modulatory, feedback signaling [primarily NMDA (49)]. Finally, it requires the coincident activation of diffuse neuromodulatory systems, such as the noradrenergic system, which occurs during salient events in wake.

#### 4.2.1 The dangers of synaptic potentiation in sleep

This last requirement implies that synaptic potentiation should occur primarily in wake, when an organism interacts with its real environment, and not in sleep, when it is disconnected and exposed to its own dreams or “fantasies” (80). While in wake non-declarative skills can be acquired under the control of environmental feedback, new learning during sleep could easily drift onto inappropriate modes. These negative consequences of synaptic potentiation during sleep are schematically illustrated in the simulations of Section 3.2.4 and 3.1.6 for declarative and procedural memories, respectively. The silence of diffuse neuromodulatory systems during most of sleep (50, 51), the associated lack of induction of genes implicated in synaptic potentiation (56, 81), and consistent evidence that true learning is not possible in sleep (82) are in line with the idea that as a rule the acquisition of new memories should be confined to wake.

It must be emphasized that the learning rule used in the present simulations is only meant to be representative of the particular mechanisms implemented by any given neuronal class. In fact, these are likely to differ, even substantially, in different species, brain structures, cell types, and developmental times (40). What the synaptic homeostasis hypothesis explicitly predicts, however, is that wake is bound to lead to a net increase in synaptic strength, which in turn needs then to be renormalized by sleep.

### 4.3 Synaptic down-selection in sleep

According to the synaptic homeostasis hypothesis, synaptic renormalization has positive effects both at the cellular level and at the systems level (38–40). At the cellular level, it leads to the restoration of energy efficiency, space, and cellular stress. At the systems level, it leads to the memory benefits investigated in the present simulations. But why should proper synaptic renormalization require sleep? The main reason has to do with statistics.

#### 4.3.1 The dangers of synaptic renormalization in wake

During a typical wake period, when the brain is on-line and learning, it is necessarily faced with a limited sampling of the statistical structure of the environment. For example, one may make a new acquaintance with whom one spends a large portion of the day. As a consequence, many neurons in one’s brain will detect and learn, primarily by synaptic potentiation, several new “suspicious coincidences” having to do with that person’s face, voice, posture, and so on, forming new memories that are potentially very useful. Under these circumstances, however, it would be maladaptive if neurons were to satisfy the requirement for renormalization by depressing those synapses that were not involved in signaling the new acquaintance, just because they were not activated much on that particular day, thus progressively erasing old memories – those of old friends – that are just as useful. These negative consequences of synaptic renormalization in wake, due to the inevitably limited sampling a given waking day affords, were illustrated by a simple example in Section 3.4.2. Conversely, as illustrated in Section 3.4.1, this problem is solved when the brain disconnects from the environment in sleep; decoupled from the requirements of behavior, the brain can afford to perform a comprehensive sampling of all its previous knowledge (old memories). In this way, connections conveying important old memories will be protected, and so will those conveying new memories that fit best with the old ones, whereas only connections conveying spurious coincidences will be at a competitive disadvantage and be depressed.

As was mentioned in the Methods, various synaptic rules enforcing depression during sleep can be envisioned, including a proportional scaling down of all synapses (16) and a rule biasing depression to spare stronger synapses more than weaker ones (48). The down-selection rule implemented here is almost a mirror image of the activity-dependent rule used for synaptic potentiation during wake (Figure 1B). This rule, too, is only meant to be representative, and in fact, as indicated in the Section “Materials and Methods,” it can be modified in various ways while leading to qualitatively similar effects. Altogether, the down-selection process ensures the survival of those circuits that are “fittest,” either because they were strengthened repeatedly during wake, or because they are better integrated with older memories. Instead, synapses involved in circuits that were only occasionally strengthened during wake, or fit less well with old memories, are depressed and eventually eliminated. As shown by the present simulations, the same rule can account for several of the beneficial effects of sleep on memory, including consolidation, gist extraction, and integration.

### 4.4 Memory consolidation

Substantial evidence exists that after sleep, compared to an equivalent period of wake, previously acquired skills (non-declarative) or facts (declarative) are retained better – an effect usually referred to as memory consolidation. This evidence is especially strong for declarative memories (1–7), but many examples of sleep-dependent enhancement of both perceptual and procedural memories exist (8–15), often with an associated local increase in sleep slow waves [e.g., Ref. (11, 83, 84)]. In some cases, the suppression of sleep slow waves has been shown to prevent the memory benefits of sleep (84, 85), while their enhancement has positive effects (25, 26).

Section 3.1.4 and 3.2.2 showed how wake-training followed by sleep-dependent down-selection in simple neuronal networks models, endowed with the learning rules of Figure 1, resulted in an enhancement of performance when re-testing after sleep, inline with experimental results obtained with both non-declarative and declarative tasks. The models illustrate that performance enhancements can be obtained without any further strengthening of connections during sleep-related spontaneous activity (so-called reactivation), but simply through a competitive protection of well-learned associations against spurious associations that were preferentially depressed. In other words, the enhancement was due to an increase in S/N, without the need for an absolute increase in the strength of connections conveying the signal. Of note, the increase in S/N after sleep led to an absolute improvement in performance in the “non-declarative,” sequence-learning task, which in the model was implemented in a segregated “channel”-like architecture. By contrast, in the “declarative,” paired associate task, implemented in an associative memory-like architecture, a period of sleep limited the decay in performance produced by an equivalent period of wake, but did not result in an absolute improvement. This difference, which is in line with a large body of experimental evidence, is explained in the model by the greater opportunities for interference during post-training wake in large associative networks.

The improvement in performance and S/N after sleep-dependent depression of synapses is also in line with previous modeling work in which different depression rules were employed. Olcese et al. implemented a large-scale model of the corticothalamic system that learned a sequence using an STDP paradigm (48). Modeling a network of Hodgkin–Huxley-like spiking neurons, Olcese et al. trained their network to reproduce a sequence of activations across four patches of neurons and demonstrated that the S/N was significantly increased when the training session was followed by synaptic renormalization during sleep. During sleep, the plasticity mechanism was modified to predominately depress connections, resulting in a selective depression of weaker synapses, while stronger synapses were preferentially protected. Just like the present simplified model (Section 3.1.3), the corticothalamic model showed preferential reactivation of the learned sequence in sleep. Moreover, this reactivation declined over time, matching experimental evidence (21, 31). Hill et al. (16) similarly compared the results of the non-declarative rotation-learning paradigm in a behavioral study and a computational model. The results of the behavioral study demonstrated that performance was enhanced in proportion to the amount of SWA during sleep, while the results of the computational model showed that sleep-dependent downscaling of synaptic weights matched the results of behavioral experiment.

In recent studies, it has been shown that after learning associations between sounds and spatial locations (69) or odors and spatial locations (68), actively cuing the encoded memories in sleep (by sound or odor, respectively) enhances memory retention. This general cuing effect has also being replicated in our simulations (Section 3.2.5). Specifically, memories cued during sleep were recalled better than without cuing, and the effect was specific, in that in the same session the recall of uncued memories did not improve. Once again, the mechanism through which selective cuing can lead to selective memory improvements after sleep (in relative terms) is that, by increasing beyond the spontaneous level the activation of specific circuits, and not of others, cued synapses are all-the-more protected from depression and have a competitive advantage over non-cued ones.

Finally, as shown in Sections 3.1.5 and 3.2.3, while spontaneous activation and synaptic down-selection in sleep were able to improve performance, additional activation through training in wake was not, and beyond a point, it actually degraded performance. This effect is accounted for in the model by the fact that the potentiation of the signal ends up saturating, and extra training mostly potentiates the noise, thereby decreasing S/N. Similar results were obtained in the simulations of Hill et al. (16), and are consistent with experimental results in perceptual learning (86–88).

### 4.5 Gist extraction and the integration of new with old memories

The brain has a remarkable ability to extract high-level invariants from sensory inputs and form gist memories, a process that is thought to be facilitated by sleep (5, 17). The simulations in Section 3.3.1 demonstrate how sleep-dependent synaptic down-selection can achieve gist extraction in a hierarchically organized neuronal network. In this network, the convergent divergent arrangement of feedforward and feedback connections leads to the more frequent spontaneous activation of top-down rather than bottom-up sequences of firing. This observation is in line with the more frequent origin of sleep slow waves in anterior rather than posterior cortices (89, 90). It is also in line with the evidence suggesting that cognitive activity during sleep is more akin to imagination than to perception (91). In the simulations, the end result of preferential top-down activation during sleep is that memory traces formed in higher areas are comparatively better protected than those formed in lower areas. In essence, the more frequent top-down activation provides an endogenous equivalent of sensory cuing (Section 3.2.5), which leads in turn to the preferential preservation of high-level, invariant features (the gist) as compared to low-level details.

Another prominent feature of memory is that new material is better remembered if it fits with previously learned schemas (73); that is, if the new memories can be integrated or incorporated with an organized body of old memories (92). Again, sleep seems to facilitate this process (5, 17). In Section 3.4.1, the potential role of activity-dependent synaptic down-selection on memory integration was examined with hierarchically organized networks of neurons. During sleep, when the old memories became spontaneously activated (which they did frequently), they also co-activated those new memories with which they had many points of contact (and vice versa). In this way, the activity-dependent down-selection mechanism protected both sets of memories from synaptic depression. However, when overlap between the old and new memories was minimal, synapses corresponding to the newly formed memory were comparatively more depressed, as they were less frequently co-activated with old memories. Moreover, they did not contribute to selecting which old memories would be best protected. Thus, as shown at an elementary level by these simulations, sleep allows new memories to interact with a large body of old memories, be consolidated depending on the extent to which they fit in their overall organization, and possibly even contribute to memory reorganization. Recent evidence indicates that learning can affect the local intensity of sleep slow waves [e.g., Ref. (11, 83, 84)] as well as their origin (93), that many slow waves are local rather than global and involve varying subsets of cortical areas in varying combinations (94), and that they travel in varying directions (89). Based on these findings and the results of the present simulations, it would seem that the joint activation of new and old memories during sleep, associated with a competitive down-selection process, not only favors their integration, but may also lead to a complex systems-level reorganization of memory.

## 5 Conclusion

Several models of memory consolidation have assumed that some of the beneficial effects of sleep on memory may occur because, in the course of NREM sleep, activity patterns acquired during learning in wake are actively replayed, leading to further potentiation of the underlying synapses. The synaptic homeostasis hypothesis argues instead that the core function of sleep is an overall renormalization of synaptic strength to counteract the progressive increase associated with learning during wake. Such renormalization would be beneficial both at the cellular and at the systems levels. While the effect on the absolute strength of synapses supporting specific memories remains unknown, molecular, electrophysiological, and structural markers indicate that, on average, there is a net decrease in connection strength throughout the cerebral cortex and the hippocampus. An overall reduction in synaptic strength during sleep is of course compatible with the occurrence of synaptic potentiation in select circuits, perhaps highlighted by tagging specific synapses during learning. However, as was pointed out above, allowing for general synaptic potentiation in sleep, when the brain is disconnected from the environment, is potentially dangerous. Moreover, it may be just as dangerous to renormalize synapses during wake, when the brain can only sample a small fraction of its memories, those that are relevant to its current situation. As was shown using simple simulations of integrate and fire neurons, a process of activity-dependent down-selection of synapses during sleep could account for several memory benefits, from consolidation of declarative and non-declarative memories, to gist extraction and the integration of new with old memories, all without the risk of degrading old memories in wake, or forming spurious ones while asleep.

## Conflict of Interest Statement

The authors declare that the research was conducted in the absence of any commercial or financial relationships that could be construed as a potential conflict of interest.
